# Artificial plasticenta: how polystyrene nanoplastics affect *in-vitro* cultured human trophoblast cells

**DOI:** 10.3389/fcell.2025.1539600

**Published:** 2025-02-24

**Authors:** Antonio Ragusa, Loredana Cristiano, Pierluigi Di Vinci, Giuseppe Familiari, Stefania Annarita Nottola, Guido Macchiarelli, Alessandro Svelato, Caterina De Luca, Denise Rinaldo, Isabella Neri, Fabio Facchinetti

**Affiliations:** ^1^ Department of Obstetrics and Gynecology, Maggiore Hospital Carlo Alberto Pizzardi, Bologna, Italy; ^2^ Section of Human Anatomy, Electron Microscopy Laboratory “Pietro M. Motta”, Department of Anatomy, Histology, Forensic Medicine and Orthopaedics, Sapienza University, Rome, Italy; ^3^ Department of Medical and Surgical Science of Infant and Adult, University of Modena and Reggio Emilia, Modena, Italy; ^4^ Department of Life, Health and Environmental Sciences, University of L’Aquila, L’Aquila, Italy; ^5^ Department of Experimental and Clinical Medicine, Magna Grecia University of Catanzaro, Catanzaro, Italy; ^6^ Department of Obstetrics and Gynecology, Magna Grecia University of Catanzaro, Catanzaro, Italy; ^7^ Department of Obstetrics and Gynecology, ASST Bergamo Est, Bolognini Hospital, Seriate, Italy

**Keywords:** placenta, plastics, polystyrene (PS) nanoplastics, altered fetal programming, confocal and transmission electron microscopy, ultrastructure

## Abstract

**Background:**

In the human placenta, we have detected the MPs by Raman microspectroscopy analysis and, for the first time, with transmission electron microscopy. MPs fragments have been localized in different compartments of placental tissue, free in the cytoplasm and within organelles like lysosomes. Moreover, their presence has been correlated with ultrastructural alterations of some cell organelles, typical of metabolic stress, mainly dilated rough endoplasmic reticulum and numerous swollen electrodense mitochondria, as well as signs derived from involuting organelles. As a result, we have speculated that microplastics in the placenta could be responsible for pathological traits activation such as oxidative stress, apoptosis, and inflammation causing long-term effects on the health of the mother and child. To demonstrate the cytotoxicity of PS-NPs on the placenta and confirm the *in vivo* results, we performed *in vitro* experiments on a trophoblast human cell line, the HTR8/SVneo cells.

**Materials and Methods:**

HTR8/SVneo cells were treated, for 24 h and 48h, with increasing concentrations (10, 25, 50, 75, and 100 μg/mL) of 0.05 µm polystyrene (PS) and cellular viability was evaluated by Counting Kit-8. Fluorescent PS-NPs examined under fluorescence/confocal microscopy were used to investigate the internalization of plastics in the placenta cells. Transmission electron microscopy was used to evaluate possible PS-NPs-dependent ultrastructural alterations of cells and organelles.

**Results:**

Our study shows that starting from 24 h exposure, PS-NPs treatment, at 50 μg/mL dose, has a cytotoxic effect on placental cells, causing the death of 40% of cells and affecting the morphology of the surviving cells. In addition, PS-NPs alter the ultrastructure of some organelles in the surviving cells, like those we have already described *in vivo*. We found that NPs enter the cells, affecting the endoplasmic reticulum and mitochondria morphology, accumulating as aggregates within lysosome-like organelles. Interestingly these aggregates become larger as the concentration of NPs increases. We speculated that the accumulation of NPs inside lysosome-like organelles could result from a prolonged and impossible attempt by the cell to remove and destroy PS. This would lead to ER and mitochondrial stress, impairing mitochondria/ER functions and oxidative stress, thus activating the apoptotic pathway and suggesting that PS-NPs could act as a cell stressor, leading to the death of cells. In support of our hypothesis, we also found NPs associated with morphological signs of cellular regression and degeneration, such as the presence of a highly vacuolized cytoplasm, dilatation, and vesiculation of ER, associated with the uncoupling/loss of associated mitochondria, cytoplasmic fragments, and free organelles deriving from cellular lysis.

**Conclusion:**

Based on electron microscopy and immunofluorescence analysis and *in vitro* study, we demonstrate the cytotoxicity of PS-NPs in trophoblast cells together with ultrastructural alterations associated with cellular regression and degeneration typical of metabolic stress. An abnormal amount of NPs in the cells might determine a persistent cellular alarm CDR (cell danger response), the evolutionarily conserved metabolic response that protects the cells and hosts from harm triggered by chemical (as in the case of NPs/MPs), physical, or biological agents that exceed the cellular capacity for homeostasis. This *in vitro* study could further help to demonstrate that the inevitable exposure of MPs/NPs in the environment, which characterizes the modern world, might be partially responsible for the epidemic of non-transmissible disease.

## 1 Introduction

**FIGURE 1:**
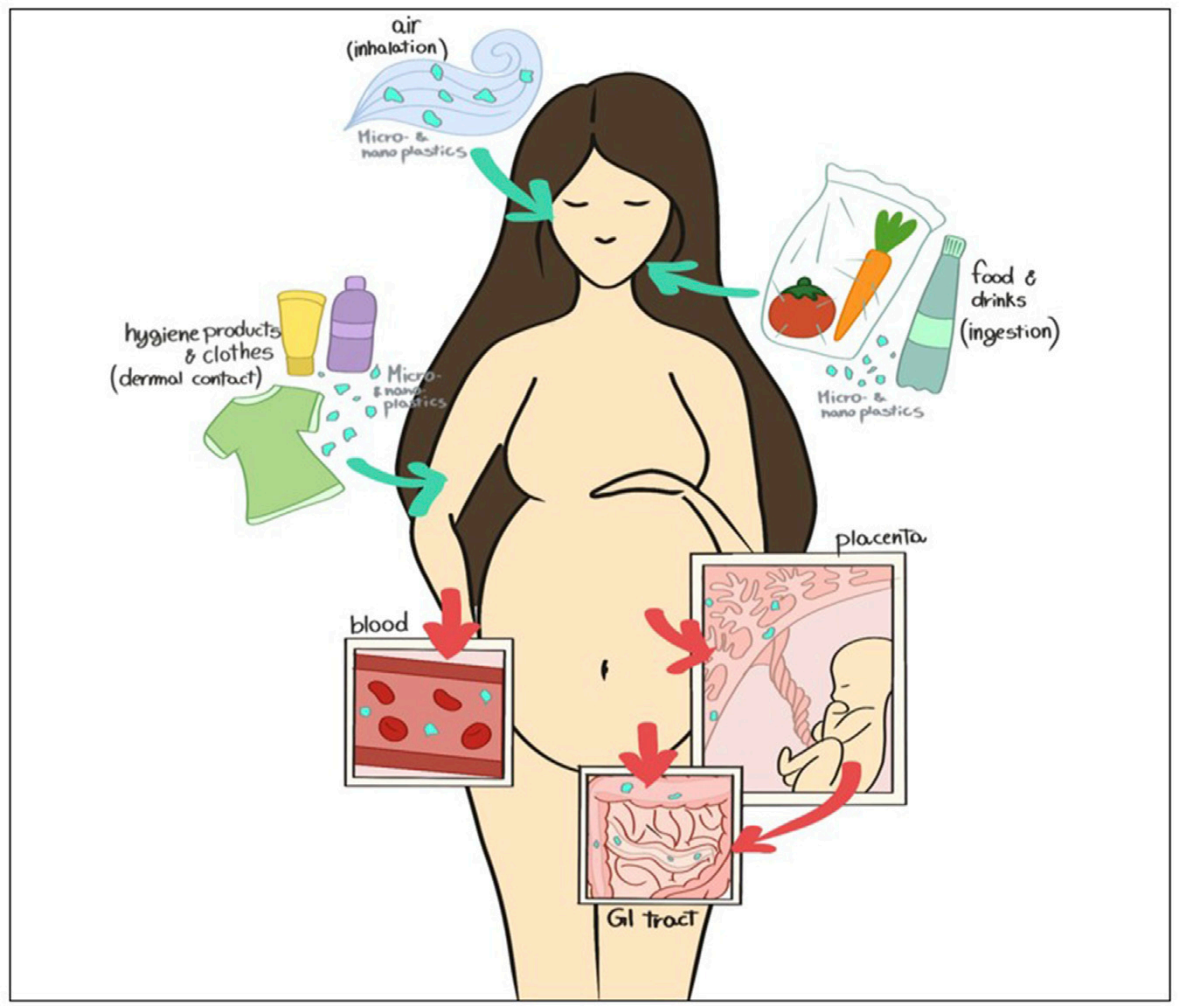
Schematic representation of NPs and MPs contamination during pregnancy (“Image Reprinted under terms of the CC-BY license (Ragusa et al., 2022c)”).

In the last century, the global production of plastics has grown exponentially to reach over 350 million tons per year, most of which pollutes the environment (GESAMP, 2016). The uncontrolled production of plastic led to excessive plastic waste. Plastics in the environment are exposed to continuous processes like photo-oxidation, chemical weathering, mechanical forces, and biodegradation activities, which affect their structural integrity and result in the fragmentation of plastic components (Hidalgo-Ruz et al., 2012; Kubowicz and Booth, 2017; Amato-Lourenco et al., 2020). It is well known that commonly used plastics, such as polyethylene (PE), polypropylene (PP), polystyrene (PS), and polyethylene terephthalate (PET) undergo a prolonged degradation process. For instance, plastic bags, commonly used in everyday life, can last 500–1,000 years in ecosystems and our bodies if we live long enough… Plastic debris refers to microplastics (MPs), defined by the European Food Safety Authority (EFSA) as plastic particles smaller than 5 mm. Particles smaller than 100 nm are defined as nanoplastics (NPs) (Hartmann et al., 2019; Chamas et al., 2020). MPs/NPs can enter human bodies via three main routes: gastrointestinal, inhalation, and dermal contact (Figure 1). Among them, ingestion is considered the major source of contamination, and recent studies estimated that a person intake from 39 to 52 thousand MPs per year (Prata et al., 2020; Cox et al., 2019). The potential health impairment caused by the internalization and accumulation of MPs is of prime concern. Although little is known about this topic, several recent studies reported evident toxic effects in animal models, marine organisms, and human cell lines (Danopoulos et al., 2022; Han et al., 2022; Yin et al., 2021; Bay et al., 2024). Once reaching the human body, MPs/NPs can cross cell membranes (Ragusa et al., 2021; Alimba et al., 2021; Avio et al., 2015); this is followed by accumulation or tentative elimination by the onset of specific cellular mechanisms. In organic tissues, MPs/NPs are not inert as previously supposed, but they are considered foreign bodies by the cells and trigger local or systemic immunoreactions (Sun et al., 2023). Moreover, particles can be the carriers of chemicals, including environmental pollutants and plastic additives, increasing the risk to human health (von Moos et al., 2012; Hartmann et al., 2019; Capriotti, 2020; Hu et al., 2022). Importantly, MPs/NPs and the compounds they contain can act as endocrine disruptors, altering normal functions of the endocrine system and causing damage to the entire organism, its progeny, or to a specific cell population of the organism itself (Lim, 2021). Animal studies confirmed the toxic effects of plastic particles on offspring generation, which interfere with cellular energy production and lipid metabolisms, leading to oxidative stress and neurotoxic response, suggesting mitochondrial dysfunctions (Jeong et al., 2022).

Since the placenta interfaces between mother and fetus, perturbation of placental functions may result in a range of adverse pregnancy outcomes such as malformation, fetal growth retardation, spontaneous abortion, and stillbirth (Lunghi et al., 2007). The placenta also plays a central role in fetal programming and adult health (Kwon and Kim, 2017).

In human placentas, MPs were detected, for the first time, by transmission electron microscopy (TEM) and correlated with ultrastructural alterations of some cell organelles in placental tissue (Ragusa et al., 2022a) to suggest possible metabolic stress as indicated by the observation of a dilatated ER, numerous electrodense swollen mitochondria and whorled membranous bodies derived from involuting ER and other structures. During trophoblast syncytialization, ER stress causes autophagy to participate in the organelle reorganization/degradation required after the incorporation of the cytoplasmic content of the fusing cytotrophoblast into the syncytium (Bastida-Ruiz et al., 2019). ER stress affects numerous processes in pregnancy (Michalak and Chan Gye, 2015; Meusser et al., 2005; Bueter et al., 2009).

**FIGURE 2:**
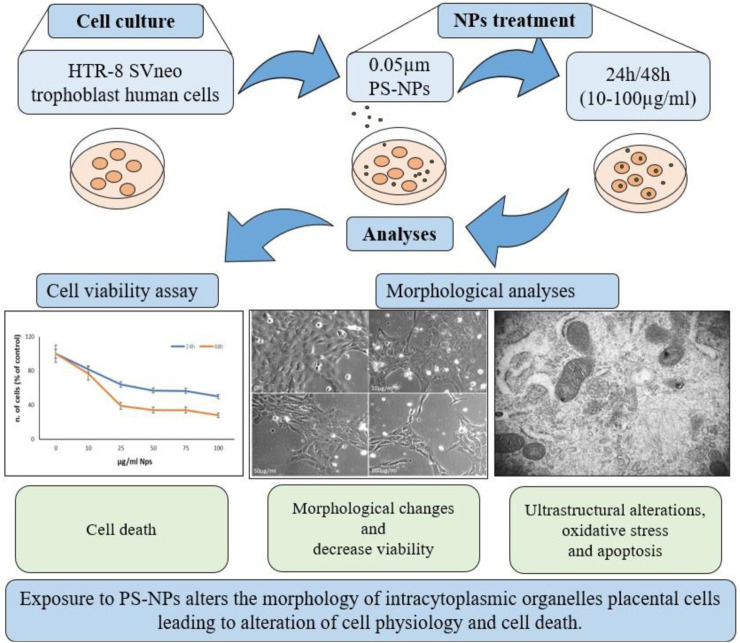
Schematic representation of study design. HTR-8 SVneo trophoblast cells were treated for 24–48 h with different concentrations of 0.05 µm PS-NPs and analyzed for cell viability and morphological alterations by light, confocal, and TEM microscopy.

This study aims to investigate the *in vitro* effects of PS NPs on trophoblast cells in terms of viability and ultrastructural analysis, mainly by TEM (Figure 2), to confirm the above results *in vivo* in the human placenta. We demonstrated that PS NPs can enter the cell, accumulating as aggregates in organelles resembling lysosomes, and act as cellular stressors by altering the structure and function of organelles and leading to cell death. So, we speculate that NPs in placental cells may cause long-term effects on human health.

## 2 Materials and methods

### 2.1 Cell cultures

HTR-8 SVneo trophoblast human cells were purchased from (ATCC, CRL-3271, Molsheim, France), cultured in adhesion, in RPMI 1640 medium (61870044, Life Technologies, CA, United States) supplemented with 1% vol/vol antibiotics (100 IU/mL penicillin, and 100 μg/mL streptomycin) and 5% Fetal bovine serum Qualified hi (FBS, 10500064, Life Technologies) and maintained, in a humidified air containing 5% CO2, at 37°C. The complete growth medium was renewed thrice weekly, and these cultural conditions were maintained throughout the study.

### 2.2 NPs treatment

To study the possible effects of PS-NPs on placental cells, trophoblast cells were seeded in 96 multiwell plates and treated for 24 h with increasing doses (10, 25, 50, 75, and 100 μg/mL) of 0.05 μm PS-NPs (08691 Polybead Microspheres, Polysciences Inc., PA, United States). To avoid a possible aggregation of the microspheres, before the treatment, a small aliquot of PS-NPs suspension was dispersed in a serum-free medium, in a glass tube, and sonicated in a bath-type sonicator (for three cycles of 30 s), according to the Manufacturer’s instructions. PS-NPs were also vortexed in repeated short pulses (5 s) and rapidly pipetted to each dilution. The treatment was performed 24h and 48 h post-seeding, replacing the medium with a freshly prepared serum-free medium containing the different doses of NPs since the presence of serum in the cell culture medium governs the internalization mechanism of neoplastic *in vitro* (Xu et al., 2019). Control cells were cultured without the addition of polystyrene microplates.

### 2.3 Cell viability assay

The effects of PS-NPs on HTR-8 SVneo cell viability were evaluated by Cell Counting Kit-8 (CCK-8, Sigma-Aldrich, St. Louis, MO, United States). Cells were seeded in 96-well plates (5,000 cells/well) with 200 μL of growth medium and were grown for 24 h in a humidified incubator with 5% CO2 at 37°C. The culture medium was removed after 24 h and 48 h and 100 μL of freshly prepared serum-free medium containing the appropriate concentration of PS-NPs (10, 25, 50, 75, 100 μg/mL). After 24 h of treatment, 10 μL of the CCK-8 solution was added to each well, incubating the plate for a further 4 h in the incubator at 37°C. The absorbance correlated to the number of living cells was measured using a microplate reader (Thermo Scientific Multiskan FC, ThermoFisher Scientific, Bothell, WA, United States) at 450 nm. CCk-8 assay was performed in triplicate. In addition, cell viability was also evaluated by cell counting with Tuerk′s solution (Sigma-Aldrich) in the Burker chamber in triplicate.

### 2.4 Immunofluorescence

To evaluate the intake of PS-NPs into the cells, the treatment was performed using 0.05 μm Fluorescent PS-NPs (17149 YG Fluoresbrite Microparticles, Polysciences Inc.**)** as described above (see Section 2.2). Cells were grown in the complete growth medium on glass slides in a 6-well plate for 24 h. The culture medium was removed after 24 h and substituted by a freshly prepared serum-free medium containing the appropriate concentration of PS-YG Fluoresbrite NPs (10, 50, 100 μg/mL). After 24 h treatment cells were washed with phosphate-buffered saline (PBS) fixed with 4% paraformaldehyde (Sigma-Aldrich) for 10 min, at RT, rewashed with PBS, and incubated with DAPI (0.5 μg/mL, Sigma-Aldrich) for cell nuclei staining. Finally, cells onto coverslips were mounted with Vectashield Mounting Medium and examined to fluorescence (Zeiss Axio Imager M2, Oberkochen, Germany) and confocal (Leica TCS SP5 II, Wetzlar, Germany**)** microscopes. Negative controls were performed by omitting PS-NPs. As positive controls, coverslips with the Fluoresbrite^®^ YG Microspheres alone were analyzed at 100 μg/mL concentration.

### 2.5 Trasmission electron microscopy

For transmission electron microscopy analysis 24 h-treated cells were detached from plates and fixed in suspension with 2.5% glutaraldehyde (Electron Microscopy Sciences, EMS, Hatfield, PA, United States) in PBS for at least 48 h, at 4°C. After several washing in PBS, trophoblast cells were post-fixed with a 2% osmium tetroxide (EMS) for 2 h, dehydrated through an ascending series of alcohol, immersed in propylene oxide (Sigma-Aldrich, St. Louis, MO, United States) for 40 min, and left overnight in a 1:1 solution of propylene oxide/epoxy resin (Agar Scientific, Stansted, United Kingdom) (first resin). The first resin was removed, and samples were embedded in epoxy resin (Agar Scientific, Stansted, United Kingdom) alone for 48 h at 60°C. Resin blocks were cut with a diamond knife in ultrathin) sections (90–100 nm using a Ul-tracut E ultramicrotome (Leica EMUC6, Wetzlar, Germany). Ultrathin sections were mounted on 100-mesh copper grids (Assing, Rome, Italy), contrasted using Uranyless (Uranyl acetate alternative) (TAAB Laboratories Equipment Ltd., Aldermaston, United Kingdom) and lead citrate (Electron Microscopy Sciences) and analyzed using a TEM (Zeiss EM10, Oberkochen, Germany), operating at 60 kV. Images were acquired using a digital camera (AMT CCD, Deben United Kingdom Ltd., Suffolk, United Kingdom) (Ragusa et al., 2022a; Miglietta et al., 2023; Relucenti et al., 2010).

### 2.6 Characterization of PS NPs

An aliquot of PS-NP stock solution was characterized by Transmission Electron Microscopy (TEM) and used as a positive control. Before observation, PS-NPs were sonicated, incubated on copper grids for 10 min at RT, washed with MilliQwater, and dyed with 2% Uranyless, according to (Gonzalez-Caballero et al., 2024).

### 2.7 Statistical analysis

The data from the different experiments were analyzed with GraphPad Prism software version 9.5.1 (GraphPad Software, LLC, San Diego, CA, United States). The values were expressed as the mean ± SEM. Comparisons between multiple groups were analyzed by ANOVA, and comparisons between two groups were performed using a T-test. Differences in values were considered significant at p < 0.05.

## 3 Results

**FIGURE 3:**
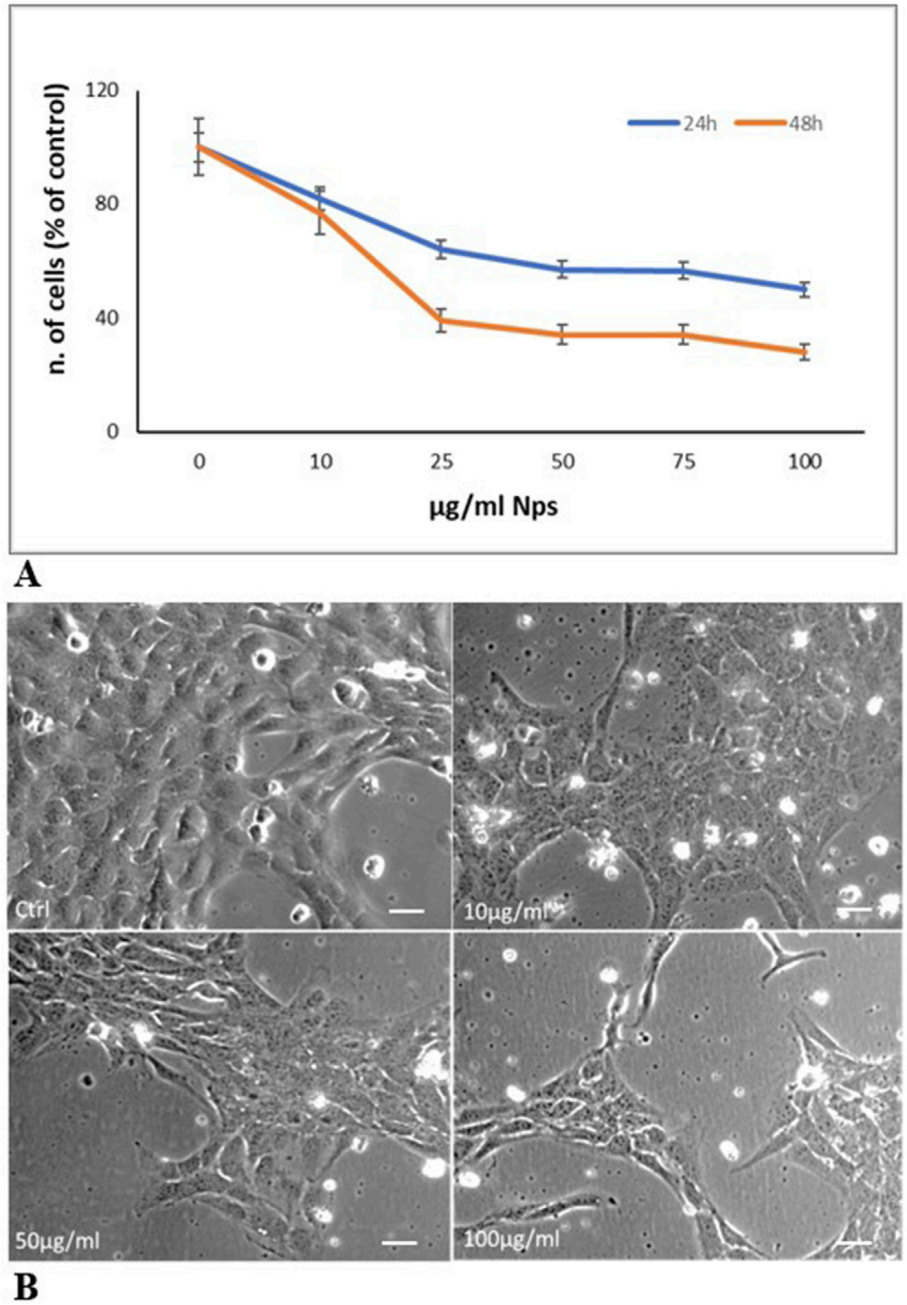
PS-Ps treatment of trophoblast cells **(A)** Cell viability evaluation with CCK-8 assay. Cells were treated with increasing concentrations of 0.05 µm NPs (10, 25, 50 75, and 100 μg/mL). Reported data represent the mean ± SD of three experiments. The differences in values compared to the control are significant (p < 0.05 at 10 μg/mL; P < 0.01 at 25–100 μg/mL) **(B)** Representative contrast phase images of cells after 24 h of treatment. Cells treated for 24 h with 10, 50, and 100 μg/mL microspheres decrease their proliferation and undergo morphological changes becoming flatter and longer and losing contact with each other. Bar, 25 µm.

Figure 3A shows the effects of PS-NPs on HTR-8/SVneo cell viability evaluated by CCk-8 assay. PS-NPs treatment, for 24 h and 48 h at different concentrations (10, 25, 50, 75, and 100 μg/mL) causes a dose and time-dependent cytotoxic effect inducing at 50 μg/mL the death of about 40% of cells, at 24 h, and more than 60% at 48 h. Figure 3B shows the decrease in cell number associated with cell morphological changes after 24 h of treatment at doses 10, 50, and 100 μg/mL. Compared to control cells, treated cells become flatter and longer, and lose contact with each other. These results suggest that environmental contaminants as NPs have a concerning role in initiating a cell damage response in human trophoblast cells.

**FIGURE 4:**
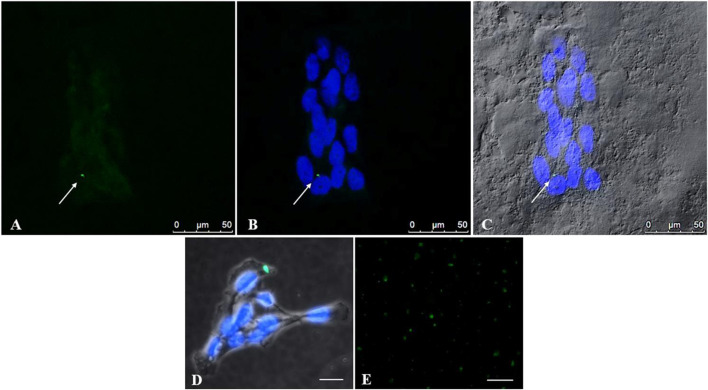
Uptake of PS-NPs by placental trophoblast cells detected with confocal microscopy. To investigate the intake of PS-NPs inside cells, trophoblast cells were treated with PS-YG Fluoresbrite NPs, for 24 h, at a 50 μg/mL dose **(A, B)** Representative microphotographs at a confocal microscope indicate the presence of aggregates of NPs (green) inside the cells. Cell nuclei were stained with DAPI (blue) **(C)** The cytoplasm of cells photographed in Differential Interference Contrast (DIC, grey) at a confocal microscope **(D)** Representative image, at fluorescence microscope, of an NPs aggregate (green), in trophoblast cells stained with DAPI. Bar, 25 µm (see attached Figure 5) **(E)** Coverslips with the Fluoresbrite^®^ YG Microspheres alone at 100 μg/mL concentration were used as positive controls. Bar, 50 µm **(A–C)**. Bar, 25 µm **(D, E)**.

Figure 4 demonstrates the presence of PS-YG Fluoresbrite NPs inside cells after 24 h of treatment, at a concentration of 50 μg/mL, under observation to confocal and fluorescence microscopes. Since the NPs size (0.05 µm) is below the confocal microscope’s resolution limit, we believe that the NP’s positivity to (green) corresponds to possible aggregates of NPs, as further evidenced by the fluorescence microscope (Figure 4D). This accumulation of NPs might cause the morphologic alteration observed in the previous data (Figure 3B). Interestingly, the NPs intake already occurs after 24 h although NPs aggregates are not necessarily detected in all cells. Moreover, PS-NPs are localized in the cell cytoplasm and not in the nuclei.

**FIGURE 5:**
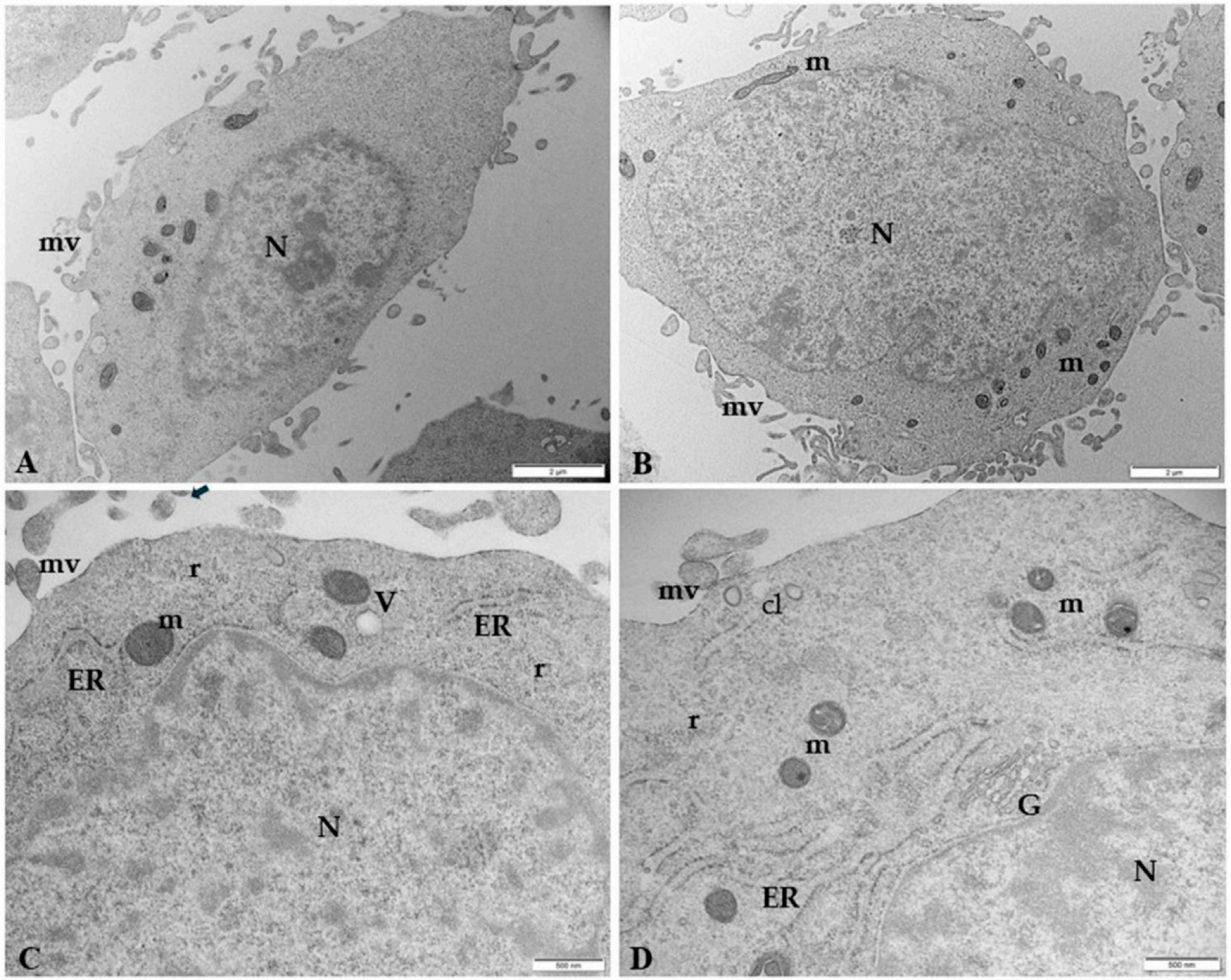
Microphotography of ultrathin sections of human control trophoblast cells by TEM **(A, B)** Low magnification of HTR-8 SVneo cells morphology. The cells are irregularly rounded, with oval large nuclei (N) and less abundant cytoplasm. Numerous blebs and microvilli (mv) are present on the surface. Bar, 2 µm **(C, D)** Ultrastructural details at higher magnification of mitochondria (m) in association with tubular vesicles of ER, clathrin vesicles (cL) under the plasma membrane, vacuoles (V), Golgi membranes (G), and polyribosomes (r). Bar, 500 nm.

Figure 5 illustrates the ultrastructural characteristics of human trophoblast cells by TEM. Placental cells are irregularly rounded cells with blebs and microvilli on the surface, exhibiting large oval nuclei with one or more nucleoli and different organelles dispersed in the scanty cytoplasm. The presence of mitochondria, close to abundant tubular cisternae of rough endoplasmic reticulum (ER), is worth noting in the cytoplasm. Clathrin-coated vesicles under plasma membrane, Golgi membranes, and vacuoles also occur in the cytoplasm.

**FIGURE 6:**
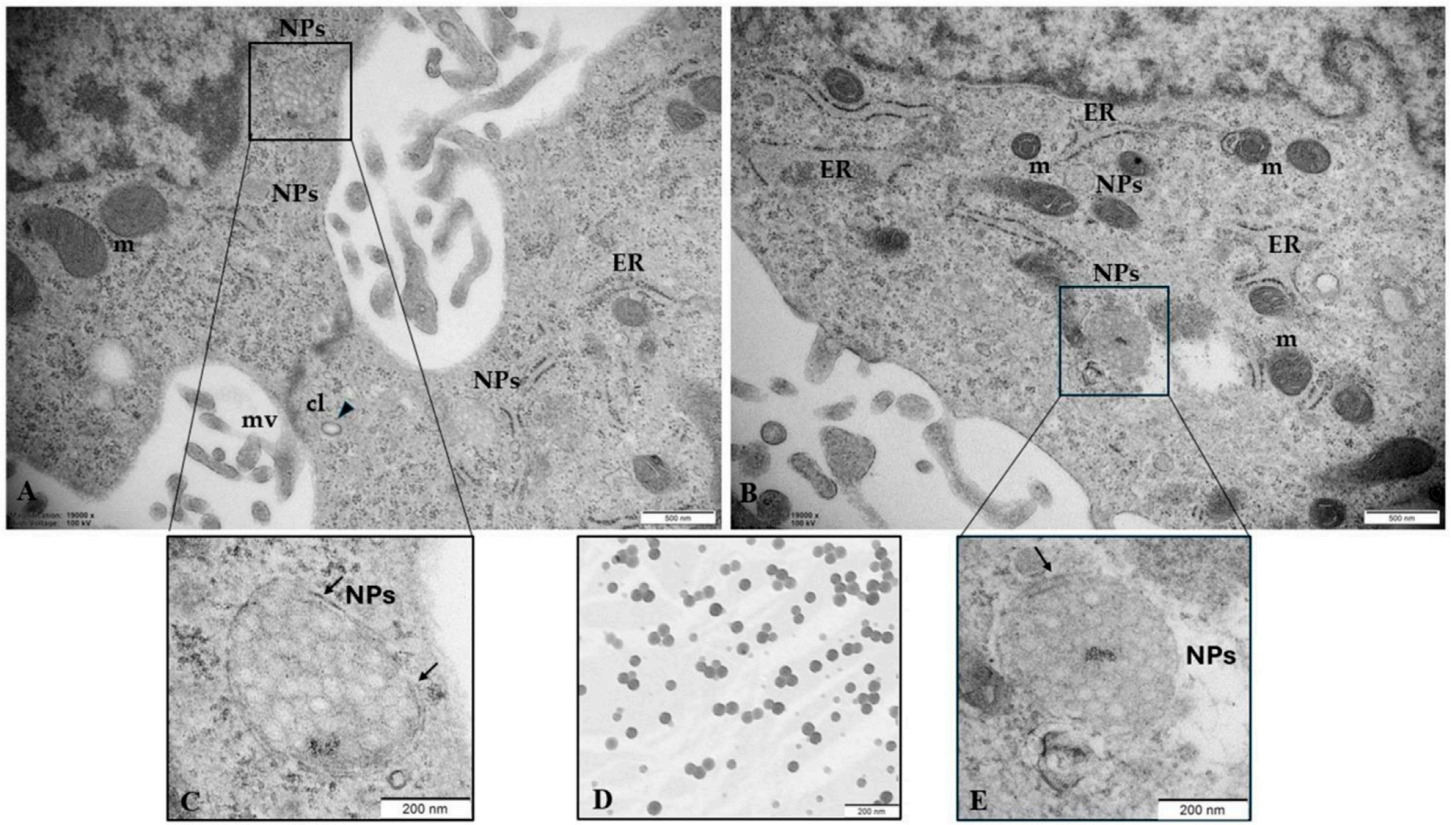
NPs in human trophoblast cells **(A, B) NPs** (50 μg/mL) were able to enter the cells, probably via endocytosis and **(C, E)** accumulate in structures constituted by a double membrane (arrows) resembling lysosomes **(D)** PS-NPs in cell medium (100 μg/mL) observed by TEM were used as a positive control, confirming the commercial particle size of 0.05 µm. Bar, 200nm. CL, clathrin vesicles; m, mitochondria; mv, microvilli. Bar, 500 µm **(A, B)**. Bar = 200 µm **(C–E)**.

Fluorescence results demonstrated that at 50 and 100 μg/mL 0.05µm, YG Fluoresbrite NPs can enter cells, forming visible aggregates of approximately 200 nm, corresponding to the instrumental resolution limit (Figure 4). The analysis of ultrathin sections of trophoblast cells by TEM (Figure 6) confirms the presence inside the cells of aggregates of NPs, encapsulated within lysosomal-like structures surrounded by a double membrane, as the result of an attempt to remove and destroy the plastic particles from the cell. Clathrin-coated vesicles (arrow point) in subplasmalemma position and close to NPs aggregates could also indicate that NPs enter cells probably via endocytosis. Moreover, the size of aggregates is enhanced with increasing NPs concentration.

**FIGURE 7:**
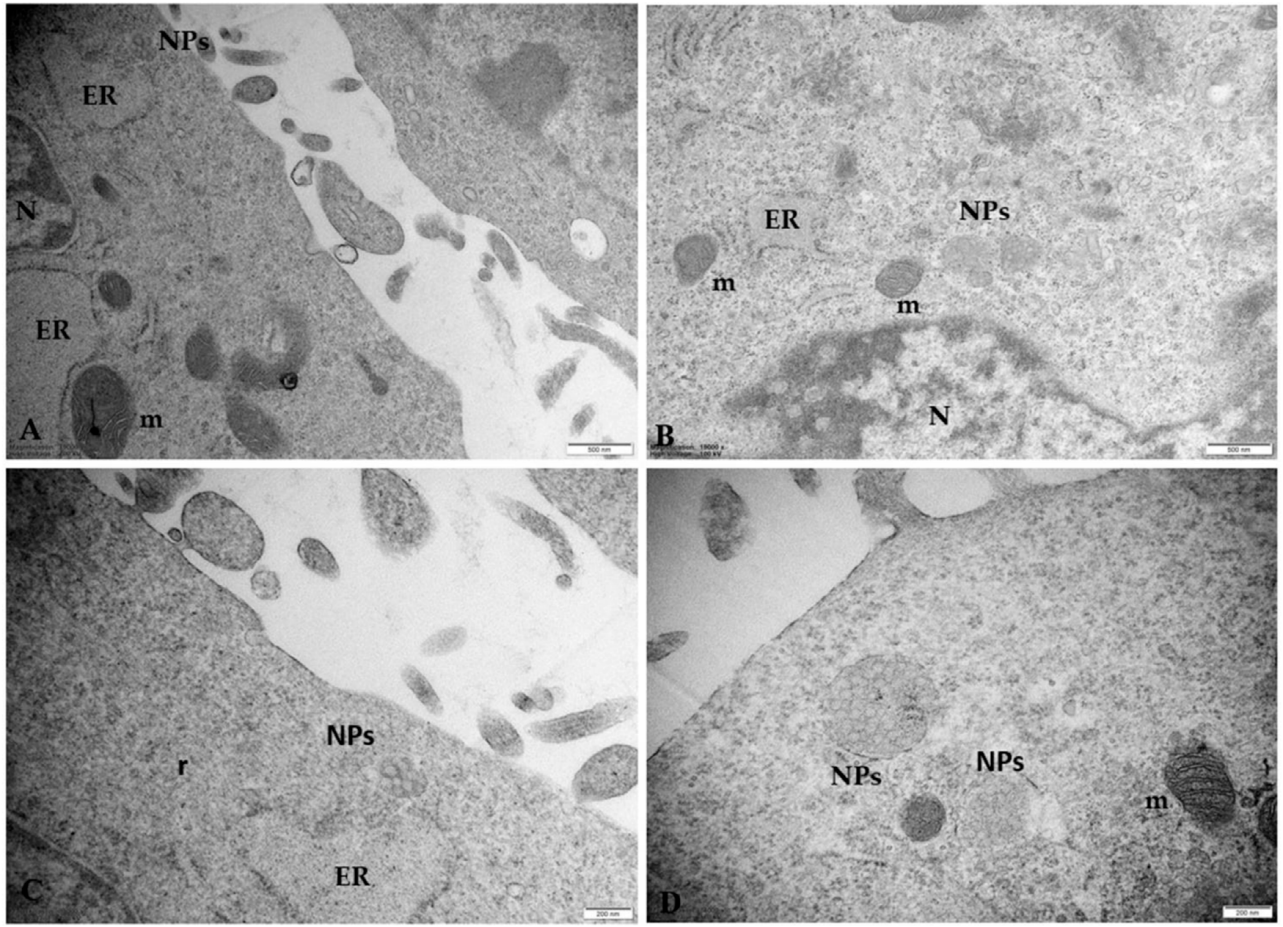
Microphotographs of trophoblast cells treated with 50 μg/mL NPs **(A–C)** NPs aggregates induce morphological changes in ER and mitochondria. The ER appears dilated, with sparse ribosomes on the outer surface. Degranulation and disaggregation of polyribosomes (r) free in the cytoplasm also occur **(D)** Microphotographs of placental cells treated with 100 μg/mL NPs in which swollen electrondense mitochondria (m) appear in the cytoplasm. Note that the size of aggregates is enhanced by increasing NP concentration. Bar, 500 nm **(A, B)**; Bar, 200 nm **(C, D)**. N, nucleus.

Figure 7 highlights the close correlation between NPs and ultrastructural alterations of the endoplasmic reticulum (ER) in cells treated with 50 μg/mL NPs. We observed aggregates of NPs close to highly dilated ER vesicles containing a moderately electrodense secretory material in the lumen and swollen mitochondria to indicate a possible NPs-induced effect of metabolic stress. These *in vitro* results confirm our previous morphological *in vivo* results on the human placental tissue.

**FIGURE 8:**
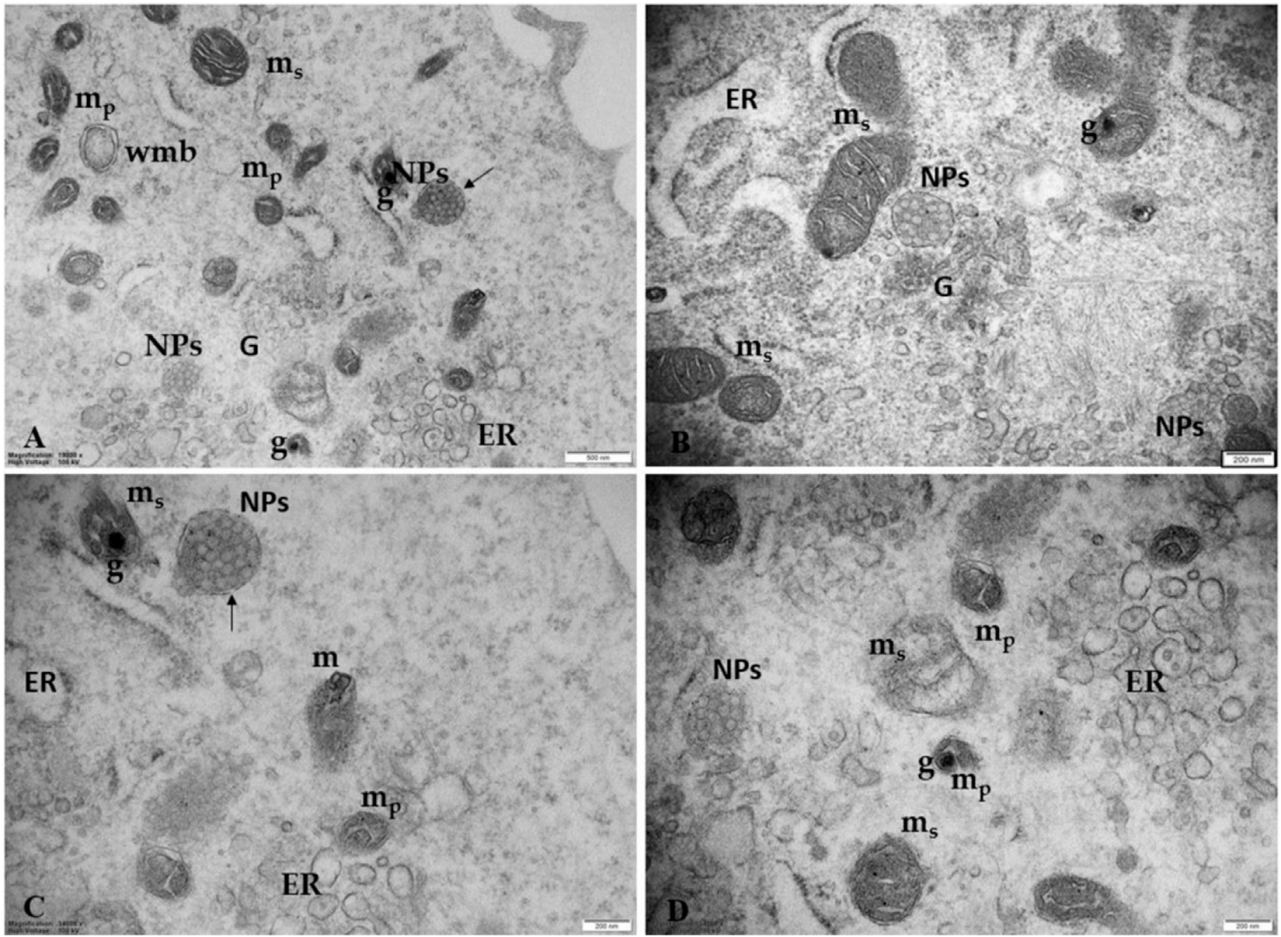
Ultrastructural changes in trophoblast cells treated with 100 μg/mL PS-NPs **(A)** PS NPs, encapsulated in a lysosome-like double membrane structure occur (arrows) in the cytoplasm together with numerous smaller, pycnotic, and electrodense mitochondria (m_p_) with prominent electron-dense granules (G), swollen mitochondria (m_s_) and numerous highly dilated ER vesicles. Bar, 500 nm **(B–D)** Morphological details at higher magnification correlating the presence of NPs with ultrastructural changes of ER and mitochondria suggest a strong turnout in support of the hypothesis of an NPs-induced possible metabolic stress. Bar, 200 nm. Golgi membranes (G).

Figure 8 shows the ultrastructural changes in trophoblast cells treated with 100 μg/mL PS-NPs. In particular, besides numerous highly dilated ER vesicles and swollen mitochondria, we also observe smaller, pycnotic, and electrodense mitochondria with prominent electron-dense granules. Although granules in the mitochondria are physiologically present, observing prominent electron-dense mitochondrial granules could also indicate a possible influx/accumulation of calcium because of mitochondrial dysfunction. This morphological framework could indicate a prolonged attempt by the cells to remove and destroy the plastic particles captured by lysosomes (autolysosomes) unable to degrade them, suggesting a possible activation of pathological pathways, such as oxidative stress and apoptosis (Figure 7).

**FIGURE 9:**
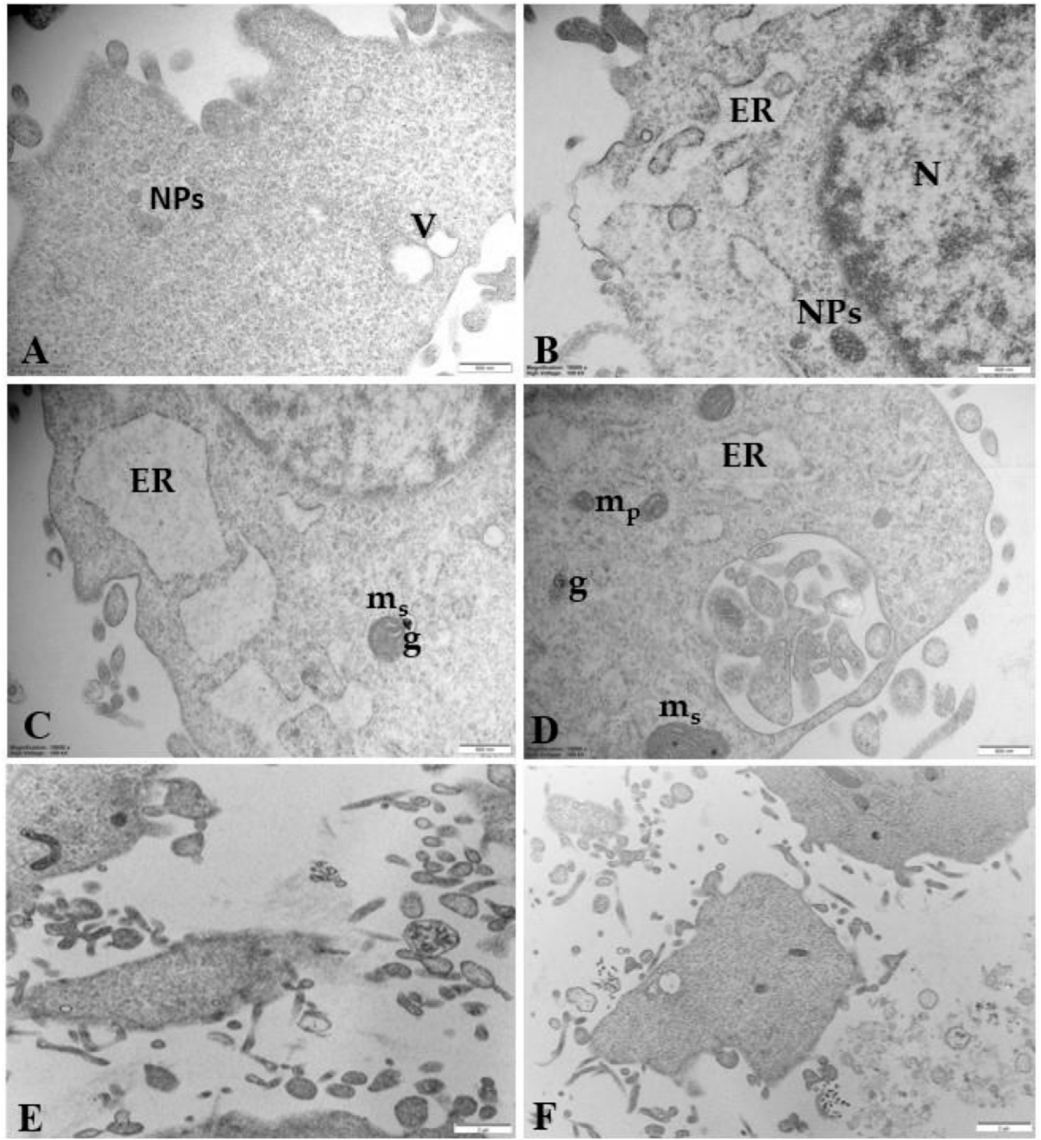
Regressing and degenerating trophoblast cells treated with 100 μg/mL PS-NPs **(A)** vacuoles (V) in the cytoplasm **(B–D)** highly vacuolized cytoplasm derived from swelling and coalescence of ER vesicles and swelling mitochondria (m_s_) with prominent electron-dense granule (G) **(E, F)** Cytoplasmic fragments and matrix-free organelles deriving from cellular lysis also occur. Bar, 500 nm **(A–D)**; Bar, 2 µm **(E, F)**.

Figure 9 highlights details of regressing and degenerating traits in trophoblast cells treated with 100 μg/mL PS-NPs. Numerous regressing elements, such as vacuolized cytoplasm and involuting organelles, are observable. Cytoplasmic fragments and matrix-free organelles deriving from cellular lysis also occur.

## 4 Discussion

The present study demonstrated, mainly at the microscopic scale, the *in vitro* presence of PS-NPs aggregates in human trophoblast cell cultures, confirming our previous *in vivo* studies on the human placenta (Ragusa et al., 2022a). We found that PS-NPs already have 24 h toxic effects on human trophoblast cells leading to 40% cell death at 50 μg/mL concentrations.

NPs’ cytotoxicity was demonstrated in many human cells: keratinocytes, intestinal epithelial cells, gastric epithelial cells, and umbilical vein endothelial cells (Ding et al., 2021; Gopinath et al., 2021; Cortés et al., 2020; Lu et al., 2022). In particular, Ahamed and Akhtar, 2024 showed that PS-NPs generate cytotoxicity in human umbilical vein endothelial cells in a concentration- and time-dependent manner and Gonzalez-Caballero et al. (2024) found that 30-nm PS-NPs treated neural stem cells undergo decreased cell proliferation and cell death by apoptosis. In human lung epithelial cells, PS-NPs of diameter 25–70 nm are rapidly internalized, significantly affecting the viability and cell cycle and inducing apoptosis (Xu et al., 2019).

By immunofluorescence results and TEM analysis, we demonstrate that within 24h, PS-NPs are taken up by cells, probably via endocytosis, as suggested by clathrin-coated vesicles under the membrane. MPs/NPs can potentially infiltrate cells directly, typically employing endocytosis mechanisms, such as channels or transport proteins, phagocytosis, macropinocytosis, as well as clathrin and caveolae-mediated endocytosis (Yee et al., 2021). Although the nature of the vesicles that envelop nanoplastics inside the cell cytoplasm is still unknown, clathrin-mediated endocytosis is one of the most important mechanisms for nanoparticle internalization and PS nanoparticles can enter cervical epithelial (HeLa), lung epithelial (A549), brain astrocytoma, and macrophage (J774A.1) through the clathrin-mediated endocytosis pathway (Guan et al., 2023). Clathrin-coated vesicles subsequently lose the clathrin coat and fuse with early endosomes, which can mature or convert into late endosomes that become competent to fuse with lysosomes (Klumperman and Raposo, 2014; Rennick et al., 2021).

In support of this suggestion, the morphological analysis, of ultrathin sections of PS-NPs-treated trophoblast cells, by TEM, also revealed that PS-NPs accumulate in lysosome-like structures surrounded by a double membrane and located close to mitochondria and ER. These results align with Huang et al., 2015, demonstrating the internalization and colocalization of 40-nm green fluorescence nanoparticles with cellular organelles including endosomes, lysosomes, mitochondria, and microtubules. In addition, other studies showed the accumulation of particles in the cytoplasm and within lysosomes, resulting in direct cellular damage attributed to inflammation and oxidative stress (Yee et al., 2021). Interestingly we found that the PS-NPs aggregates inside structures surrounded by a double membrane, in the cytoplasm, become larger as the concentration of NPs increases close to organelles morphologically altered.

In fact, not less significant is the observation concerning the association between the presence of these aggregates and the development of ultrastructural alterations typical for metabolic stress, mainly found in the ER and mitochondria. We observed a dilated rough ER and/or partitioned ER into many vesicles close to swelling mitochondria with ballooning cristae and pycnotic mitochondria with prominent electron-dense granules in their matrices. Although granules in the mitochondria are physiologically present, observing prominent electron-dense mitochondrial granules could also indicate a possible influx/accumulation of calcium because of mitochondrial dysfunction. This morphological framework could indicate a prolonged attempt by the cells to remove and destroy the plastic particles captured by lysosomes (autolysosomes) unable to degrade them, suggesting a possible activation of pathological pathways, such as oxidative stress and apoptosis. Our data are in line with Gonzalez-Caballero et al. (2024), who reported, by observation under TEM, the *in vitro* uptake of 30 nm PS NPs in human neural stem cells hNS1, their aggregation in an “accumulating structure,” early endosomal vacuoles/vacuoles-like, together to morphological changes of hNS1 cells and apoptosis. Changes in the mitochondrial ultrastructure and mitochondrial swelling were also observed by Liu et al. (2022) in mouse spermatocyte GC-2 cells and by Ding et al. (2021) in gastric epithelial GES-1 cells after treatment with PS-NPs.

Moreover, our results fully confirm our previous *in vivo* study on placental tissue where MPs fragments, detected in the basement membrane, the syncytiotrophoblast and cytotrophoblast, pericytes, and endothelial cells surrounding the fetal capillaries, free in the cytoplasm and inside lysosome-like organelles, were correlated with ultrastructural alterations of some cell organelles in placental tissue, mainly the ER and mitochondria (Ragusa et al., 2022a).

These altered morphological features could be compatible with a cell stress phenotype (Palladino et al., 2021).

Therefore, we confirm the hypothesis that PS-NPs could act as a cell stressor, leading to the death of cells. The significative morphological alterations we found in the trophoblast cells could result from a prolonged attempt by cells to remove and destroy NPs, as demonstrated by the fact that PS-NPs, once they enter the cell, are captured by organelles/vesicles resembling lysosomes, unable to degrade them since PS-NPs are indestructible. This would lead to ER and mitochondrial stress, impairing mitochondria/ER functions and oxidative stress, thus activating the apoptotic pathway (Ragusa et al., 2022a). So, mitochondria and ER are thought to play a crucial role in the cellular response to NPs accumulation. Also, Sousa et al. (2024), in placental explants, demonstrated that PS-NPs could cross the placental barrier, possibly through endocytosis, and induce significant cytotoxicity, oxidative stress, and metabolic changes. In addition, in the NPs-treated earthworm *Eisenia fetida*, NPs are gradually internalized by earthworm coelomocytes and amassed in lysosome-like structures. Those agglomerations stimulate lysosomal membranes to lose stability and even rupture, inhibiting the autophagy process, cellular clearance, and, eventually, coelomocyte death. NPs presence is also associated with decreased antioxidant enzyme activity and reactive oxygen, causing pathological abnormalities (Zhou et al., 2023).

In support of our hypothesis, we also found NPs associated with morphological signs of cellular regression and degeneration, such as the presence of a highly vacuolized cytoplasm, dilatation, and vesiculation of ER, associated with the uncoupling/loss of associated mitochondria, cytoplasmic fragments, and free organelles deriving from cellular lysis. Thus, the metabolic stress induced by PS-NPs could be responsible for the ultrastructural alterations associated with cellular regression and degeneration. In the mouse placenta, Huang et al. (2015) found that nanoparticles can cross the placenta and induce trophoblast apoptosis.

An abnormal amount of NPs in the cells might induce a persistent state of cellular alarm, particularly the cell damage response (CDR), the evolutionarily conserved metabolic response that protects cells and hosts from harm. It is triggered by encounters with chemical (as in the case of NPs/MPs), physical, or biological threats that exceed the cellular capacity for homeostasis (Naviaux, 2020) and cause cell death due to the inability of the cells to degrade them. “When the CDR persists abnormally, whole body metabolism and the gut microbiome are disturbed, the collective performance of multiple organ systems is impaired, behavior is changed, and chronic disease results…” *(* Naviaux et al., 2016).

Neurotoxicity signals, leading to a decline in cognitive, learning, and memory abilities, were observed in hippocampal neuronal cells of the offspring from female rats, gavaged throughout gestation and lactation with low PS NPs dosages (Chen et al., 2024).

Dramatic changes in the expression of genes involved in immune response, inflammation, neurodegenerative and cardiovascular diseases, and lipid metabolism (Shi et al., 2024) were demonstrated by transcriptomic and metabolomic analyses in spleen tissue samples of female mice watered for 49 days with different concentrations of 0.1 μm PS NPs.

Unexpectedly and intriguingly, polystyrene, in association with a hyperlipidemic diet, can induce obesity and a change in the microbiome of the mouse intestines. Moreover, the transplantation of feces between mice subjected to PS-MPs exposure and a high-fat diet and control mice can change metabolic expression (Zhai et al., 2024). These mechanisms are most likely also active in humans and could be one of the reasons for the obesity epidemic currently underway around the world (Chooi et al., 2019).

Although we believe our findings are important for maternal and newborn health, two limitations occur in our study that could be a bias in the analysis: the first is represented by using plastic tools (pipets, tips, well plates, media bottles) to perform *in vitro* studies; the second is the use of only polystyrene plastic while plenty of plastic fragment’s type occurs in the environment. Pregnancy is exposed to NPs contaminants, and the placenta is known to be affected by different types of plastic accumulation, as previously demonstrated through Raman microspectroscopy analysis (Ragusa et al., 2021). Moreover, in a recent study, Wieland et al. (2024) highlighted that nominally identical particles from various manufacturers differ in their ζ-potential (electrical potential of a particle in a medium on its sliding plane) to interact with cells and their internalization probability. The environmental exposure of microplastic particles can alter their ζ-potential and, consequently, their internalization probability. In addition, Shen et al. (2022) have shown that PS-NPs of different sizes and surface charges had a specific toxicity pattern on human placental cells that depended on size and surface charge. The smaller the size of the PS-NPs, the greater the toxicity induced on human placental cells in terms of oxidative stress increase, DNA damage, inflammation, and apoptosis. Therefore, choosing the microplastic model can give different results in NPs/MPs effect studies (Brown et al., 2001).

Our study demonstrates the stressful effect of PS-NPs, leading to damage and even death, in human placental cells cultured *in vitro* confirming our previous *in vivo* study (Ragusa et al., 2022a). NPs in the human maternal body can interfere with cell biology and physiology, influencing signaling pathways crucial for cell activities, such as cell proliferation, morphology and function of organelles (mitochondria and ER), and apoptosis, in the fetus and mother.

Further researches will be conducted to investigate the effects of NPs on different primary cultures of placental cells to: 1. Correctly simulate the cellular variability of the placenta, and the variability in the shape, composition and size of microplastics; 2. Correlate any effects with women’s consumption habits of plastic products, including food and cosmetics. NPs could also influence gene expression through epigenetic alterations and investigating these aspects will improve the wellbeing of mothers and newborns.

These *in vitro* studies may further contribute to demonstrating that the unavoidable exposure of MP/NP in the environment, which characterizes the modern world, may be partially responsible for the current epidemic of non-communicable diseases.

## 5 Conclusion

The ubiquitous presence of MPs/NPs in the environment has led to unavoidable human body exposure through skin contact, inhalation, and ingestion (Ragusa et al., 2022c). In addition, our previous study demonstrated abnormal accumulation of MPs/NPs in human breast milk (Ragusa et al., 2022b), emphasizing that breastfeeding may cause the transfer of such particles to the offspring, causing eventual health problems to the child (Ragusa et al., 2021).

Developmental Origins of Health and Disease (DOHaD) explains how environmental pollutants, such as plastics if introduced in the epoch of intrauterine development or during the early years of life may alter developmental pathways, leading to maladaptive responses to environmental challenges later in life (Siddeek and Simeoni, 2022). Limited exposure to environmental pollution from plastics during intrauterine development may reverse the impact on future health.

MPs/NPs are becoming an increasingly serious public health problem, and knowing their effect on placental health will help us understand how to limit damage to the placenta and improve maternal and neonatal health.

## Data Availability

The original contributions presented in the study are included in the article/Supplementary Material, further inquiries can be directed to the corresponding author.
